# Analysis of RNA-Seq data using self-supervised learning for vital status prediction of colorectal cancer patients

**DOI:** 10.1186/s12859-023-05347-4

**Published:** 2023-06-07

**Authors:** Girivinay Padegal, Murali Krishna Rao, Om Amitesh Boggaram Ravishankar, Sathwik Acharya, Prashanth Athri, Gowri Srinivasa

**Affiliations:** 1 PES Center for Pattern Recognition, Department of Computer Science and Engineering, PES University, Bengaluru, 560085 India; 2 Department of Computer Science and Engineering, PES University Electronic City Campus, Bengaluru, 560100 India

**Keywords:** Vital status, RNA-Seq, Self-supervised learning, Cancer, Interpretability

## Abstract

**Background:**

RNA sequencing (RNA-Seq) is a technique that utilises the capabilities of next-generation sequencing to study a cellular transcriptome i.e., to determine the amount of RNA at a given time for a given biological sample. The advancement of RNA-Seq technology has resulted in a large volume of gene expression data for analysis.

**Results:**

Our computational model (built on top of TabNet) is first pretrained on an unlabelled dataset of multiple types of adenomas and adenocarcinomas and later fine-tuned on the labelled dataset, showing promising results in the context of the estimation of the vital status of colorectal cancer patients. We achieve a final cross-validated (ROC-AUC) Score of 0.88 by using multiple modalities of data.

**Conclusion:**

The results of this study demonstrate that self-supervised learning methods pretrained on a vast corpus of unlabelled data outperform traditional supervised learning methods such as XGBoost, Neural Networks, and Decision Trees that have been prevalent in the tabular domain. The results of this study are further boosted by the inclusion of multiple modalities of data pertaining to the patients in question. We find that genes such as RBM3, GSPT1, MAD2L1, and others important to the computation model’s prediction task obtained through model interpretability corroborate with pathological evidence in current literature.

**Supplementary Information:**

The online version contains supplementary material available at 10.1186/s12859-023-05347-4.

## Background

Cancer is a leading cause of deaths worldwide, accounting for nearly 10 million deaths in 2020, or nearly one in six deaths. In a multi-stage process that often goes from a pre-cancerous lesion to a malignant tumour, cancer develops when normal cells undergo a transition into tumour cells. According to research, it is presently possible to prevent between 30 and 50% percent of cancers by avoiding risk factors and using proven evidence-based preventative methods. Many cancers have a high chance of cure if diagnosed early and treated appropriately [1–4]. Fortunately, recent advancements in sequencing techniques in bio-informatics have gained traction and have given rise to studies pertaining to the identification of cancer biomarkers and quicker diagnosis of the disease. However, very little research has been done on estimating the vital status of patients diagnosed with cancer. In this study, we analyse the gene expression dataset obtained from the TCGA website, to estimate the vital status of patients in the context of colorectal cancer [5]. The vital status property is a binary variable that indicates whether the patient survived cancer. A vital status value of 0 represents a patient that survived; a vital status value of 1 represents a patient that succumbed to cancer. The outcome of this study could not only help determine a suitable course of treatment but also help in a better understanding of the disease through a study of the bio-markers that are identified as important predictors by the computational models we have built.

Owing to the tabular nature of the gene expression dataset, applications of self-supervised learning techniques on gene expression data (tabular data here) have not made it to the limelight as much as they have in the domains of Computer Vision and Natural Language Processing. This is mainly because tabular models must be able to accommodate features from different discrete and continuous distributions and uncover correlations without relying on the positional information.

So why is self-supervised learning worth exploring in the context of gene expression tabular data? Apart from the increase in performance that comes with deep learning models, it also enables fusion with multiple other modalities of data (say copy number variation and clinical data) – eliminating the need for feature engineering, representation learning, and end-to-end compositional multi-task models.

With this line of thought, our work can be summarised as follows:

- Initially, RNA Sequencing gene expression data pertaining to 519 entries is procured from The Cancer Genome Atlas (TCGA). Owing to the high dimensional nature of the dataset, we have proposed a variety of feature selection techniques to mitigate the effects of the curse of dimensionality. The features chosen are used to train classical machine learning models (such as XGBoost, and Logistic Regression) without the self-supervised learning objective.
- Subsequently, the efficacy of self-supervised learning methods in this domain is explored via TabNet. This requires the procurement of additional unlabelled data to be used for pretraining, also obtained from TCGA across various cancer projects, with the unifying factor being the type of cancer, which was selected to be “Adenomas and Adenocarcinomas”.
- The TabNet model is first pretrained on the unlabelled corpus, after which the pretrained model is fine-tuned on the labelled dataset.
- The explainability of the best-performing computational model is demonstrated by plotting its feature importance mapping. Additionally, majority of the genes deemed as important by the prediction task are in agreement with existing literature.
- In the interest of further improvement in score, additional modalities of data, namely Copy Number Variation (CNV) and Clinical data pertaining to the same patients are obtained in the same manner as above. These modalities of data then undergo preprocessing in parallel and all the modalities are joined using Submitter ID as the join key.
- These different modalities of data are trained using the TabNet model with a self-supervised learning objective and encouraging results are obtained in the context of vital status estimation.

## Related work

### Predictive modelling and associated computational methods

The earliest work on the estimation of vital status using RNA-Seq data serves as the baseline model for this study [6]. They use the LASSO model and two variants of neural networks. The results from this study establish that basic neural networks are computationally expensive and do not outperform well-established simple models like LASSO for this problem. In the context of pure machine learning-based analysis, the work of [7] talks about oversampling approaches such as SMOTE to address the class imbalance in the dataset. Using the analysis of variance (ANOVA) test as a feature reduction strategy, they demonstrate the superiority of the PanClassif model in the context of both binary and multiclass cancer prediction.

In the domain of deep learning, the work of [8] is noteworthy as the study is based on the popular transformers architecture and self-attention mechanism for the classification of Lung Cancer sub-types. The major goal of this research was to develop an end-to-end strategy for classification tasks that could handle both binary and multi-class classification issues and produces state-of-the-art results. Apart from this, the work of [9] proposes the usage of Graph Convolution neural networks for the identification of tumour and non-tumour samples (of 33 cancer categories) based on gene expression profiles. They showed that these models can achieve accurate classification (above 94%) utilizing cancer-specific marker genes using data from the TCGA dataset.

On the multimodal front, the work of [10] proposes a multimodal deep learning method for long-term cancer survival prediction. They use different model architectures for different modalities, in order to optimize results from each one, before aggregating them to obtain a consolidated result. The metric of note used is the concordance index, a way to measure the risk scores obtained from the model against ground truth survival times.

### Feature reduction of high-dimensional data

To combat the curse of dimensionality inherent in the data, there are a variety of useful analyses of feature selection techniques that can be used. A comparison of 10 dimensionality reduction techniques using 30 simulation datasets and 5 real datasets has been performed [11]. These dimensionality reduction techniques have been evaluated based on factors such as stability, accuracy, and computing cost [12]. Proposes a method that, unlike other dimension reduction strategies, employs an ensemble learning scheme and uses a large number of weak learners to perform an accurate similarity search for simultaneous reduction in dimensionality and feature gene extraction.

### Self-supervised learning

Self-supervised learning (SSL) methods have predominantly been focused on natural language processing [13, 14] and computer vision [15–17], where the concept of correlation between sequential features is at the fore. Recently, however, these self-supervised learning approaches have been introduced to work with tabular data using attention-based mechanisms [18] and random feature corruption [19], with these methods performing better than common industry favourite supervised learning methods such as XGBoost [20] and LightGBM [21, 22]. Proposes a new self-supervised learning method called TabNet that works on tabular data and is heavily referred to in this paper.

## Methods

### Problem definition

In this study, we analyse the vital status (0: survived, 1: succumbed) of those patients diagnosed with colon cancer. This study is performed in two settings – the unimodal data setting (pertaining to gene expression, hereafter referred to as the study of RNA-Seq data) and the multimodal data setting (pertaining to gene expression, copy number variation, and clinical data).

For the study of RNA-Seq data, the problem can be formulated as predicting the vital status ${\textbf {y}} \in \{0, 1\}$ given the sample’s gene expression value ${\textbf {X}} = \{{x}_{1}, {x}_{2},{x}_{3},...... {x}_{m}\}$ where m represents the number of genes after feature selection.

For the multimodal data setting, the problem can be formulated as predicting the vital status ${\textbf {y}} \in \{0, 1\}$ given the values ${\textbf {A}} = \{{a}_{1}, {a}_{2},{a}_{3}\}$ which represent the gene expression, copy number variation, and clinical data values respectively. The fusion strategy employed here is that of early fusion where the three modalities of data are concatenated together before being passed into the computational pipeline.

### Pipeline for study of RNA-Seq sata (unimodal data setting)

**Fig. 1:**
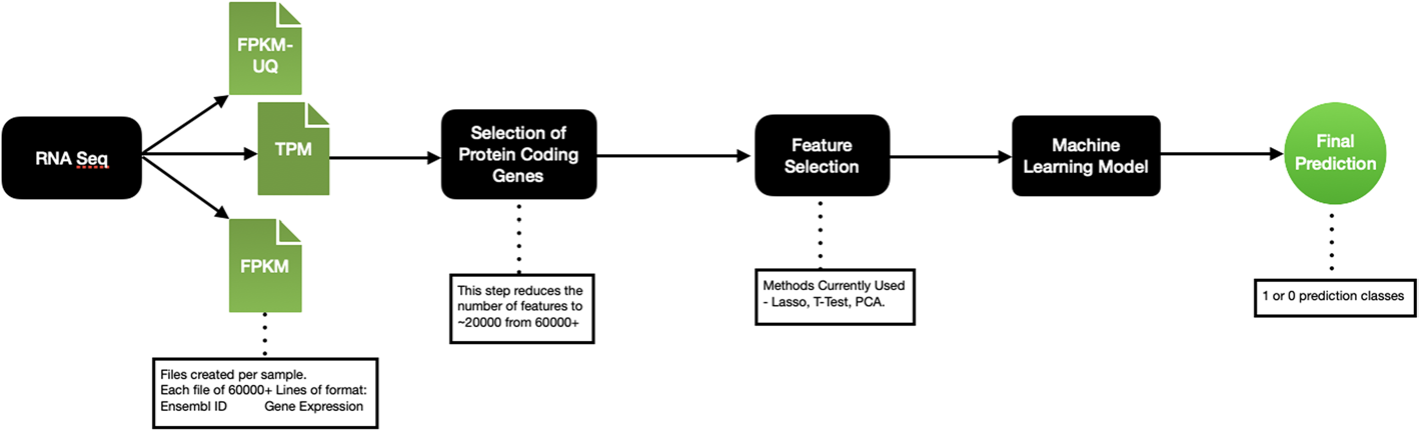
Workflow used for the supervised approaches in this study

The pipeline for the study of RNA-Seq data using supervised learning methods can be found in Fig. 1.

#### Procurement of RNA-Seq data

The labelled data used in this study pertains to the TCGA-COAD project, focusing on Colon Adenocarcinoma. This data was downloaded from the GDC data portal [23], with filters set to obtain the vital status annotation (deceased and not-deceased) for the gene expression data as well. Unlabelled data is also obtained from TCGA across various projects, with the unifying factor being the type of cancer (Adenomas and Adenocarcinomas); this was done due to the fact that COAD was majorly comprised of that particular type of cancer (85.25%) (see Additional file 1: Section SM1.1 for further details on RNA-Seq data procurement). Each of the data points in this dataset came with 3 different values for the same gene expression data: TPM, FPKM and FPKM-UQ.

##### TPM

As first defined by [24], Transcripts Per Million (TPM) has frequently been touted as a normalization technique that does not employ batch adjustment. TPM normalizes each read during a run such that the total number of readings is exactly one million. Every read count is represented by TPM as a percentage of all the other reads that are mapped to throughout the run.

##### FPKM and FPKM-UQ

Due to technical biases such as depth of sequencing and gene length, the normalization of the abundance values quantified in the previous section needs to occur in order to make gene expression values comparable within and across samples.

The fragments per kilobase of transcript per million mapped reads (FPKM) calculation aims to control for transcript length and overall sequencing quantity. The upper quartile FPKM (FPKM-UQ) is a modified FPKM calculation in which the protein-coding gene in the 75th percentile position is substituted for the sequencing quantity. This is thought to provide a more stable value than including the noisier genes at the extremes. They are computed as follows:

1 $$\begin{aligned} FPKM = \frac{C_{g} * 10^9}{\left(\sum _{i=1}^{N}C_{i}\right)L_{g}} \end{aligned}$$

and

2 $$\begin{aligned} FPKM-UQ = \frac{C_{g} * 10^9}{C_{qtl(0.75)}*G*L_{g}}, \end{aligned}$$

where *N* denotes the number of protein-coding genes, $C_\text {g}$ denotes the count of reads aligned to gene g, $L_\text {g}$ denotes the union length of exons of gene g, G denotes the number of protein-coding genes on autosomes and $C_\text {qtl(0.75)}$ denotes the counts aligned to the gene at quantile 0.75.

Upon experimentation with the above measures, this study proceeds to use FPKM-UQ as its preferred unit due to better model performance with data.

The COAD dataset initially has a total of 519 data points with 60,660 features. However, this data is reduced to 445 data points to match submitter IDs with other modalities, in order to obtain a fair comparison with the multimodal workflow. This is clearly detailed in this section.

The most glaring inference that can be made is that the dataset suffers massively from the curse of dimensionality. Table 1 represents the features of the COAD dataset as per the interest of this study:

**Table 1:** The COAD dataset

Dataset	No. data points	Alive	Deceased	No. genes (total)
COAD	445	344	101	60,660

#### Feature reduction

##### Protein coding genes

A multitude of different gene types that include coding, non-coding, and pseudo-coding exist. Based on the literature, a subset of genes known as the protein-coding genes, which are present in the human genome, are essential to the study of human biology and medicine and, as a result, have a significant impact on cancer vital status prediction. The original TCGA patient dataset had 60,660 gene ensemble IDs (features), therefore the first phase of feature reduction is decreasing that number to 19,962 protein-coding genes.

##### Computational techniques

 The second step of feature reduction is the usage of one of three different feature reduction techniques, namely -

- L1 / Lasso [25]
- Statistical t-test [26]
- Principal Component Analysis (PCA) [27]

The number of features filtered as important by each feature reduction technique can be seen in Table 2. To provide a thorough comparative study, each of these feature reduction strategies has been implemented with every machine learning and deep learning model, with an understanding of the inner workings of each methodology. Other statistically based feature selection approaches like Laplacian Score and Autoencoders were also tested, however, none of them showed as much promise as the ones described above (see Additional file 1: Section SM2).

**Table 2:** Number of features selected by each computational method

Feature reduction	No. features
Lasso	80
PCA	199
T-test	230

For Lasso, an inverse regularisation strength of 0.001 was used, with the genes with non-zero weights then selected as a filter. For PCA, the number of features captured corresponded to 99.9% of the variance across genes. For the t-test, a *p*-value of 0.01 was used as a cutoff for a feature to be selected. These values were determined experimentally as the best parameter values for the respective feature reduction methods.

This feature reduction is performed on the **labelled** dataset, with the same features used as a filter (or a transform, in the case of PCA) on the **unlabelled** dataset.

#### Supervised learning methods

A variety of predictive analyses have been conducted on the data. These, in combination with the different feature reduction techniques used, resulted in a set of methods used from start to finish to model the vital status of the patient. The workflow for the same has been elucidated in Fig. 1. The methods of predictive analysis used are described below.

##### Logistic regression

 Logistic regression is one of the most common and useful classification algorithms in machine learning. It is a supervised machine learning algorithm that can be used to evaluate the probability of a certain target value or class. In this case, it is used to model the probability of the vital status of the patient as either 0 or 1. Logistic Regression is thought to be a baseline in this work owing to its simplicity [28]. The model used an l2 penalty, with an inverse regularisation strength (i.e. inverse lambda) value of 0.001.

##### Artificial neural network (Feedforward MLP)

 ANNs are nonlinear models which discover complex relationships between the input and targets to discover inherent patterns. An artificial neural network has three or more layers; the first layer consists of input neurons; which send data into the intermediate layers, which in turn consolidate and send the final output data to the output layer. While more complicated than most other methods, the capturing of complex relationships between features is better done by the neural network. A network of 4 layers with hidden layer sizes as (64, 32, 128, and 64) respectively, and ReLU activation performed the best on this data.

##### KNN

 KNN is a non-parametric and lazy learning algorithm. In short, the algorithm entails a (usually) weighted distance calculated between the target and the learned data. The best k points are the basis for the class chosen. In the classification phase, k is a user-defined constant, and an unlabelled data point is classified by assigning the label which is most frequent among the k training samples nearest to that query point.

The inferior results obtained using PCA as a feature reduction technique was an indication that the eigenspace was a bad representation of the feature set which implies the lack of correlation within the data. This in turn spurred us to consider using KNN in order to obtain better results. Training with the 25 nearest neighbours yielded the best results.

##### Explainable boosting machines

 A Generalized Additive Model with automated interaction detection, Explainable Boosting Machine (EBM) is tree-based and boosts gradients in a cyclic fashion. The boosting process is carefully constrained to train on a single feature at a time in round-robin form with a very low learning rate, making the order of the features irrelevant. Second, pairwise interaction is automatically found and included by EBM, increasing accuracy while maintaining intelligibility. Apart from the default parameter set, tweaking the parameters max leaves (3), and max bins (40) yielded better results. This has been implemented using the Interpret ML package.

##### XGBoost

 XGBoost is a decision-tree-based ensemble Machine Learning algorithm that uses a gradient-boosting framework.

In gradient boosting, each predictor improves on its predecessor’s error; It relies on the fact that the best possible model, when combined with previously trained models, minimizes the overall prediction error. The key idea is to set the target values for the coming model in order to minimize the error. XGBoost has been used extensively with RNA-Seq data due to its tabular nature [29]. The model performed the best with features extracted by the t-test, tweaking the model with 120 estimators and a maximum depth of 3 gave better results.

#### Self-supervised learning methods

Self-supervised learning (SSL) is a machine learning technique developed to solve the challenges posed by the over-dependence of labelled data, either due to the complexities involved in finding it or simply due to the unavailability of labelled data.

The aim of this technique is to capture subtle nuances in data that do not require the presence of labels. This allows the subsequent training done on the labelled data to be more useful in the prediction task itself, as opposed to learning structures within data.

##### Data procurement

 The procurement of the labelled dataset follows the same workflow as detailed in the section above. Additionally, an unlabelled dataset consisting of the same gene IDs, with a total size of 4801 samples from various types of adenomas and adenocarcinomas is also procured by the same means for self-supervised learning purposes.

##### TabNet

 TabNet is a neural network designed to handle tabular data well as it also allows for pretraining on unlabelled data. TabNet uses sequential attention to choose features at each decision step, enabling interpretability and better learning as the learning capacity is used for the most useful features.

The TabNet architecture has an encoder and decoder module wherein the former is inspired by top-down attention using sparse instance-wise feature selection constructed on top of a sequential multi-step architecture. For the self-supervised learning objective, a decoder architecture has been proposed for reconstructing the masked features from the encoded representation. To do this a binary mask S has been chosen as ${\textbf {S}} \in \{0, 1\}^{B \times D}$ wherein the fully connected layer and feature transformers have to predict the masked feature at each decision step based on the reconstruction loss as follows:

3 $$\begin{aligned} \sum _{b=1}^{B}\sum _{j=1}^{D} {\left| \frac{({\hat{f}}_{b,j} - f_{b, j}) \cdot S_{b, j}}{\sqrt{\sum _{b=1}^{B} (f_{b, j} - 1 / B \sum _{b=1}^{B} f_{b, j})^2}} \right| }^2 \end{aligned}$$

where ${f}_{b, j}$ and ${\hat{f}}_{b,j}$ correspond to the feature importance score and the expected feature importance score of the ${j}^{th}$ feature in the $b^{th}$ sample.

Finally, TabNet also has a built-in interpretability mechanism that quantifies the contribution of each feature to the trained model across the dataset.

**Fig. 2:**
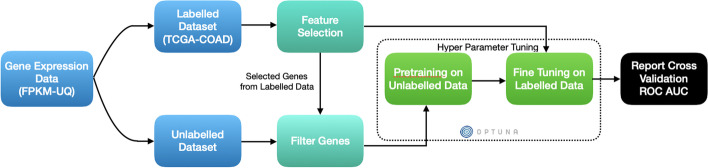
Workflow used for self-supervised learning in the unimodal data setting

The workflow, as elucidated in Fig. 2, for the above is as follows:

- In the first step, feature reduction is performed on the labelled dataset as we assume that this captures the important features.
- The same subset of features selected from the labelled dataset is filtered in the unlabelled dataset as well.
- The TabNet pretrainer is then trained on the unlabelled dataset in order to capture the embedding space of the gene expression data.
- The pretrained model is then trained on a training split of the labelled subset in a 5-fold cross-validation, the average receiver operating characteristic area under the curve (ROC-AUC) score of which is reported in the end. (This stage also employs hyperparameter tuning using Optuna [30] to search for the best set of hyperparameters that maximize the ROC-AUC score of the model on cross-validation)

### Pipeline for study of multimodal data

**Fig. 3:**
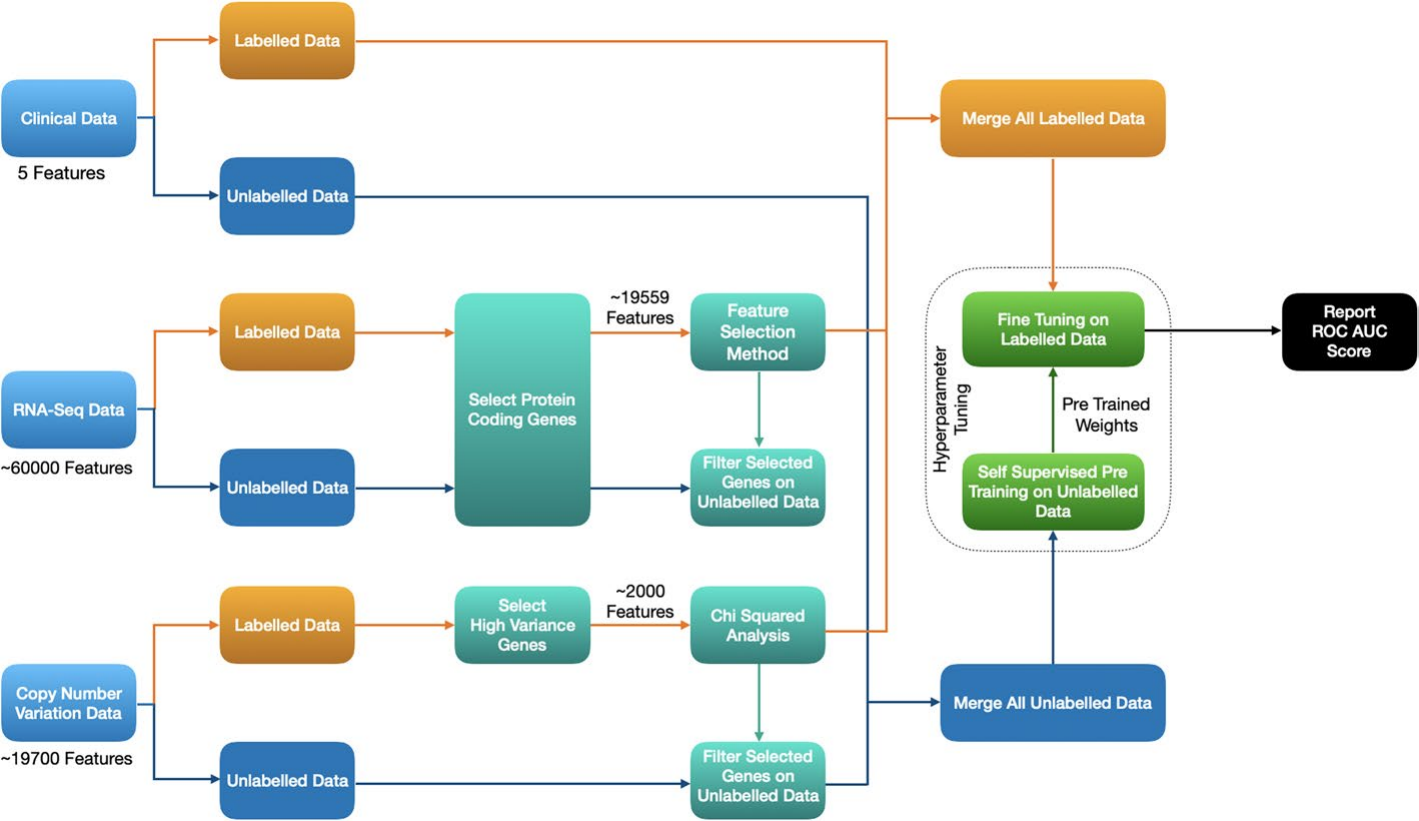
Workflow used for the multimodal data pipeline in this study

The workflow integrating the usage of multimodal data with self-supervised learning in this study is presented in Fig. 3. The details of which are as follows:

#### Procurement and preprocessing

The superior results of TabNet as compared to supervised learning methods sparked our interest in the possibility of improving the obtained results further. Two additional modalities, namely Copy Number Variation (abbreviated as CNV) and Clinical data for the same patients were downloaded from TCGA. The manifest files for the above were **obtained from** [10], and were used to procure these modalities of data. The procurement and preprocessing of RNA-Seq data follow the same pipeline as described in the pipeline for study of RNA-Seq Data (unimodal data setting). T-test, as discussed before, provides the best results among all the feature reduction techniques and is the sole feature reduction technique that is employed here.

##### Copy number variation

 Copy Number Variation captures the variation in the number of copies of a certain DNA sequence across different patients. While this is also done on a per-gene basis, this is different from gene expression (i.e., RNA-Seq). The CNV dataset consists of only categorical variables.

The first step of preprocessing pertaining to CNV data involves filtering only the high-variance genes. The genes that seem to vary more across the set of patients can be assumed to be more important and contain more information. The top 2000 highly varying genes are selected. Subsequently, Chi-Squared Analysis, which is known to be effective against categorical data, is used as the final step of feature reduction. The 256 genes (empirical) deemed most important by Chi-Squared Analysis are selected in this stage. (see Additional file 1: Section SM1.2 for more details on preprocessing CNV data)

##### Clinical data

 Clinical data refers to information on a patient’s exposures, demographics, diagnosis, family links, and laboratory testing. It provides information on influential parameters such as age, ethnicity, prior treatment, prior malignancy, etc.

Clinical data, as important as it may seem, unfortunately, has an abundance of missing values. Only features with missing values below a predefined threshold are selected and a variety of sanity checks are performed to finally arrive at a total of 5 features (see Additional file 1: Section SM1.3 for more details on preprocessing clinical data).

The final step of preprocessing involves encoding the categorical features and introducing a new class to represent missing values in each column.

##### Submitter ID matching and early fusion

 As implemented previously, in each modality, the same set of features that are filtered using the feature reduction technique in the labelled dataset are selected in the unlabelled dataset as well.

As the final preprocessing step, only those patients whose records are present in all three modalities are considered. A patient P, whose data is missing from even one modality, is discarded from the other modalities as well. Post fusion, the final unlabelled dataset is of the size (4300, 495) and the final labelled dataset is of the size (445, 496).

In order to eliminate any bias, the RNA-Seq data obtained post submitter ID matching was used in all the previous experiments.

#### Self-supervised learning in the multimodal data setting

As discussed above, the pipeline for the study of multimodal data follows the same workflow as the self-supervised workflow in unimodal data setting due to its superior results as compared to the supervised models.

The self-supervised pretraining step involves the TabNet model mapping the embedding space of the unlabelled multimodal data from which the pretrained weights are then obtained. This is then followed by a fine-tuning stage, where the pretrained weights are used to train a TabNet Classifier that is then trained on the labelled multimodal data. The Optuna framework is employed for hyperparameter search for these stages. Finally, the optimal model is used to report the average ROC-AUC score over a 5-fold cross-validation.

## Results

### Configuration and training details

All experiments carried out with the models used in this study have their scores reported after a 5-fold cross-validation over the TCGA-COAD (Labelled) dataset, using Google Colab with an NVIDIA Tesla K80 GPU.

### Results with RNA-Seq data (unimodal data setting)

This study provides a strong and thorough base on the efficacy of supervised learning approaches by combining each model with each feature reduction technique. Although we have tried a host of computational models, only the ones that show the highest scores have been enlisted below.

**Table 3:** ROC-AUC score as per feature reduction and supervised training approach

Model	Feature reduction	ROC-AUC
Lasso as per [6]	–	0.57 ± 0.05
$DeepNet_i$ as per [6]	T-Test	0.58 ± 0.04
Logistic regression	Lasso	0.721 ± 0.059
	PCA	0.512 ± 0.029
	T-test	**0.724 ± 0.026**
Neural network	Lasso	0.528 ± 0.091
	PCA	0.539 ± 0.116
	T-test	0.657 ± 0.089
Explainable boosting machines	Lasso	0.581 ± 0.075
	PCA	0.534 ± 0.06
	T-test	0.700 ± 0.056
KNN	Lasso	0.55 ± 0.09
	PCA	0.553 ± 0.089
	T-test	0.544 ± 0.051
XGBoost	Lasso	0.581 ± 0.058
	PCA	0.579 ± 0.055
	T-test	0.692 ± 0.061

The RoC-AUC of the best performing model is indicated in bold

**Fig. 4:**
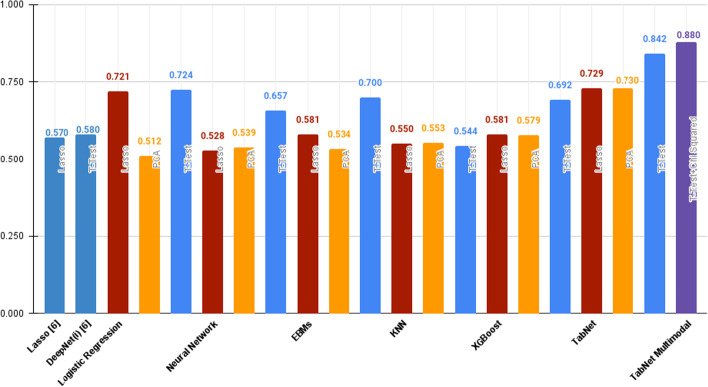
Graph of ROC-AUC scores of all models

It can be seen in Table 3 that the Logistic Regression model that uses an l2 penalty, with t-test as the feature reduction technique performs the best with a final ROC-AUC score of **0**.**742** among the various supervised learning approaches. The scores obtained by this model is also better than that obtained by [6] which serves as the inspiration for this study. Also, a general trend that can be observed in Fig. 4 is that, on average, t-test as the feature reduction technique provides the best results (the blue bars as seen in Fig. 4). This prompted us to extrapolate on this hypothesis and make use of the t-test as the sole feature reduction technique further on in the study for computational feasibility purposes.

Each model was also tested with a t-test *p*-value threshold of 0.001. In this setup, the best-performing model in particular did not see a stark change in the mean ROC-AUC but did see a rise in standard deviation in the results (see Additional file 1: Section SM3.1). This is likely due to the fact that the reduced number of features selected [28] were not as well-rounded of a representation of each random fold.

Further on in the study, we explore the efficacy of self-supervised learning approaches on the prediction of vital status. TabNet was tried using both the supervised (without pretraining) and self-supervised approaches to clearly demonstrate the performance gain obtained via pretraining. Our results, as can be seen, in Table 4, conclusively prove that the self-supervised learning approach outperforms the supervised learning approaches. The TabNet model makes use of the Adam [31] optimiser with a ReduceLROnPlateau scheduler. The metrics reported in this study pertain to the average ROC-AUC Score of the model’s performance on the labelled (TCGA-COAD) dataset.

**Table 4:** ROC-AUC score per feature reduction technique for TabNet

Model	Feature reduction	ROC-AUC
TabNet without pretraining (supervised learning)	Lasso	0.712 ± 0.032
	PCA	0.642 ± 0.054
	T-test	0.742 ± 0.042
TabNet with pretraining (self supervised learning)	Lasso	0.729 ± 0.031
	PCA	0.73 ± 0.047
	T-test	**0.842 ± 0.022**

The RoC-AUC of the best performing model is indicated in bold

A pretrained TabNet model with the employment of t-test as a feature selection technique shows the highest score of **0**.**842** obtained with RNA-Seq data (unimodal data setting). Optuna was used to tune hyperparameters such as weight decay, learning rate, patience (for early stopping), and gamma (feature reuse in masks).

### Results with multimodal data

As can be seen in Table 4, TabNet with t-test as the feature reduction technique seemingly performs the best. This prompted us to make use of only this setup (t-test + TabNet) in the multimodal workflow in order to reduce computational complexity. As can be seen, in Fig. 4, using the t-test + TabNet setup on multimodal data results in yet another boost in performance. TabNet trained with the self supervised learning objective on multimodal data provides an ROC-AUC score of **0.88 ± 0.019** in the context of vital status prediction.

## Discussion

### Interpretability

This study makes use of model interpretability, which helps quantify and visualise the effect of features in the dataset, i.e., the gene expressions to the vital status predicted by the models used. As elucidated in the previous sections, TabNet is the best-performing model in our study with a final ROC-AUC score of 0.842 on RNA-Seq data (unimodal data) and 0.88 on multimodal data. The genes deemed important by TabNet are verified in literature to bring confidence in their predictions.

TabNet consists of an encoder which is comprised of an attentive transformer, a feature transformer and a feature mask, and a decoder which is comprised of just the feature transformer. The decoder employs a sparse feature selector at each step. Differing from SHAP, which uses cooperative game theory, TabNet [22] computes the feature importances via the aggregate feature importance mask, in the following way:

4 $$\begin{aligned} {{M}_{agg-b, j} \sum _{i=1}^{{N}_{steps}}{{\eta }_{b}[i]{M}_{b,j}[i]}/\sum _{j=1}^{D}\sum _{i=1}^{{N}_{steps}}{{\eta }_{b}[i]{M}_{b,j}[i]^2}} \end{aligned}$$

In the above equation, ${M}_{b, j}[i]$ corresponds to feature importance of ${f}_{b, j}$ and ${\eta }_{b}[i]$ weighs the aggregate contribution of the ${i}^{th}$ decision step via a ReLU activation [32].

**Fig. 5:**
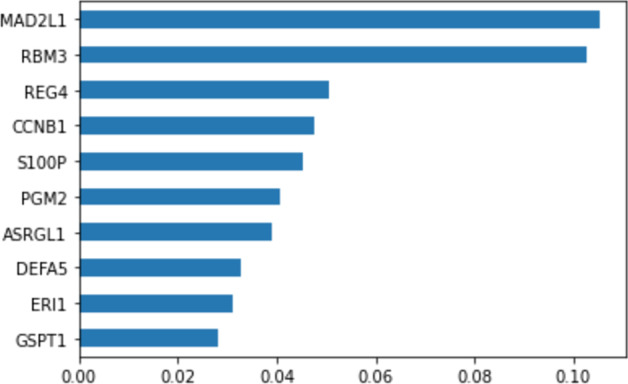
Aggregate feature importances observed in TabNet

Using the values in the aggregate feature mask of the best performing TabNet model (using the t-test feature selection technique), the feature importances as described in Fig. 5 was observed.

Similar to the approach used with supervised techniques, using the genes (features) selected in Fig. 5, a further literature survey was conducted to see if the genes deemed significant by the selected self-supervised TabNet model has any pathological evidence.

**Table 5:** Gene ID and its pathological evidence towards colon cancer as per TabNet

Gene ID	Gene name	Pathological evidence [33]
ENSG00000164109	MAD2L1	Yes [34]
ENSG00000102317	RBM3	Yes [35]
ENSG00000134193	REG4	Yes [36]
ENSG00000134057	CCNB1	Yes [37]
ENSG00000163993	S100P	Yes [38]
ENSG00000169299	PGM2	No evidence found
ENSG00000162174	ASRGL1	No evidence found
ENSG00000164816	DEFA5	Yes [39]
ENSG00000104626	ERI1	No evidence found
ENSG00000103342	GSPT1	Yes [40]

As observed from Table 5, 7 of the top 10 genes marked as significant by TabNet have pathological evidence recorded in the literature. The gene MAD2L1 has been shown to be a potential biomarker for colorectal cancer as per [34]. The study revealed that there is a higher expression of MAD2L1 in CRC cell lines and tissues. The gene RBM3 is a well-known gene in terms of prognostication of colorectal cancer. It is described in that, that over-expression of RBM3 has subsequent effects on epithelial proliferation and stemness in colorectal cancer [35]. Interestingly, the gene PGM2 has not been shown to be a biomarker for colorectal cancer while the gene ASRGL1 has been shown to be prognostic in the prediction of endometrial cancer [41]. Therefore, the findings of this project may spark an interest in researching the contribution of these genes towards the prognosis of colorectal cancer. (See SM4 for a more comprehensive set of genes and their importance.)

To further understand the results, functional enrichment analysis was performed on full list of genes, ordered by their feature importances as determined by the TabNet model. The list of pathways obtained are below:

### Pathway enrichment analysis

In this section, we look into the associated pathways with the important genes as inferred from the TabNet approach as presented above. The list of important genes with non-zero importances, in order, was selected as the input for GProfiler which yielded the results as observed in Table 6. From the aforementioned list, it is found that cell cycle G2/M phase transition is closely linked to colorectal cancer, as mentioned in [42]. However, further study on the association that pathways found in this study have to colorectal cancer can provide valuable insight and serve as a direction for future work.

**Table 6:** Pathways obtained in order of importance, from GProfiler

Source	Pathway
GO:BP	Cell cycle G2/M phase transition
GO:BP	Regulation of mitotic spindle checkpoint
GO:BP	Regulation of mitotic cell cycle spindle assembly checkpoint
GO:BP	Regulation of spindle checkpoint
GO:BP	Cell cycle phase transition
GO:CC	Nucleoplasm
GO:CC	Organelle lumen
GO:CC	Intracellular organelle lumen
GO:CC	Membrane-enclosed lumen
GO:CC	Mitotic spindle assembly checkpoint MAD1-MAD2 complex
GO:CC	Secretory granule lumen
GO:CC	Cytoplasmic vesicle lumen
GO:CC	Vesicle lumen
GO:CC	Nuclear lumen
GO:CC	Mitotic checkpoint complex
REAC	Alpha-defensins
MIRNA	hsa-miR-24-3p

### Future work

#### Survival analysis

Patients’ survival (or time to event) statistics are frequently gathered and evaluated in clinical studies. The information may be used to compare two or more groups in terms of both the overall number of occurrences and the frequency at which a particular event happens. The distribution of many other forms of data does not apply to survival data; they are non-negative and frequently skewed depending on how quickly events happen. More significantly, they frequently experience censoring (missing or incomplete data), which can happen for a number of different reasons. The work of [10] details a multimodal approach to survival analysis. The module used for the RNA-Seq modality is a fully connected (FC) neural network. Due to the exemplary performance of TabNet across this study, thought may perhaps be given to replacing the FC module with TabNet instead, to yield better performance. This could also be augmented by adopting the further modalities of data detailed in [10] and using a modified version of TabNet for every other modality.

## Supplementary Information

**Additional file 1.** SM1. Data Procurement and Processing. SM2. Other Feature Reduction Techniques. SM3. Other Results. SM4. Feature Importances. SM5. Hyperparameter Tuning.

## Data Availability

The datasets analysed in the study is available at the Genomic Data Commons (GDC) Data portal (https://portal.gdc.cancer.gov) as a part of the TCGA Program. Specifically, the RNA-Seq data used to pretrain the unsupervised models (across various projects in the TCGA program) can be found at the following link: RNA-Seq - Adenomas and Adenocarcinomas The RNA-Seq data used in the prediction task (i.e, TCGA-COAD data) can be found at the following links: RNA-Seq - COAD Vital Status: AliveRNA-Seq - COAD Vital Status: Deceased In the interest of reproducible research, all the code used in the paper for both the preprocessing and cleaning of the above data, as well as for all the experiments done has been made available online.
