# Supplementary material for: Analysis of RNA-Seq data using self-supervised learning for vital status prediction of colorectal cancer patients
Source: BMC Bioinformatics. 2023 Jun 7;24:241. doi: 10.1186/s12859-023-05347-4 (PMC10249191; doi:10.1186/s12859-023-05347-4)
Supplement: Supplementary file 1 — Additional file 1. SM1. Data Procurement and Processing. SM2. Other Feature Reduction Techniques. SM3. Other Results. SM4. Feature Importances. SM5. Hyperparameter Tuning. [file 12859_2023_5347_MOESM1_ESM.pdf]

# Analysis of RNA-Seq Data using Self-Supervised Learning for Vital Status Prediction of Colorectal Cancer Patients

Girivinay Padegal, Murali Krishna Rao, Om Amitesh B.R., Sathwik Acharya, Prashanth Athri and Gowri Srinivasa

## Supplementary Material

## Table of Contents

**SM1. Data Procurement and Processing** **2**
SM1.1 RNA-Seq data: COAD 2
SM1.1.1 Manifest File for RNA-Seq data 2
SM1.2 CNV Data 2
SM1.2.1 Manifest File for CNV Data 2
SM1.2.2 CNV Data Preprocessing 2
SM1.3 Clinical Data 4
SM1.3.1 Clinical Data Procurement 4
SM1.3.2 Clinical Data Preprocessing 4
**SM2. Other Feature Reduction Techniques** **5**
SM2.1 Laplacian Score 5
SM2.3 Autoencoders 6
SM2.4 Regularisation using Linear SVC 6
**SM3. Other Results** **7**
SM3.1 Results with a t-test p-value of 0.001 7
SM3.2 Results with different sizes of data 8
SM3.3 Multimodal Results with other models(apart from Tabnet): 8
**SM4. Feature Importances** **9**
SM4.1 Logistic Regression Model 9
SM4.2 TabNet 10
**SM5. Hyperparameter Tuning** **11**
SM5.1 Hyperparameter tuning for TabNet - RNA-Seq 12
SM5.2 Hyperparameter tuning for TabNet - Multimodal 14

# SM1. Data Procurement and Processing

The RNA-Seq data is obtained by choosing the required parameters on the Genomic Data Commons (GDC) portal, downloading the corresponding manifest file, and using the GDC Client with the manifest to download patient files [1].

## SM1.1 RNA-Seq data: COAD

### *SM1.1.1 Manifest File for RNA-Seq data*

The manifest files for both the labelled and unlabelled RNA-Seq data were obtained directly from the GDC portal.

COAD Alive [2]

COAD Dead [3]

Adenomas and Adenocarcinomas [4]

## SM1.2 CNV Data

### *SM1.2.1 Manifest File for CNV Data*

The manifest file for both the labelled and unlabelled CNV Data was procured from [5]. Using the GDC Client tool enabled us to download the files consisting of the focal scores by the gene for each of the 33 TCGA Projects available.

### *SM1.2.2 CNV Data Preprocessing*

CNV Data that was procured using the manifest file has file names as aliquot IDs that need to be mapped to their respective patient submitter IDs. This is done by procuring the Aliquot ID data from the GDC Portal. A get request is sent to the GDC server at https://api.gdc.cancer.gov/cases with the following parameters –

- ‘fields': 'cases.patients.portions.analytes.aliquots'
- 'format': 'TSV'
- 'size': '200000'

The aliquot data is stored in a dataframe called cnv_aliquotes which looks as follows –

| | aliquot_ids.0 | aliquot_ids.1 | aliquot_ids.10 | aliquot_ids.100 | aliquot_ids.101 | aliquot_ids.102 | aliquot_ids.103 |
|---|---|---|---|---|---|---|---|
| **submitter_id** | | | | | | | |
| **TARGET-50-PAJMMC** | ccf2eea7-1459-5119-a22f-7c59bacacc95 | 16c0e04c-318b-4577-8b04-8898cb239577 | NaN | NaN | NaN | NaN | NaN |
| **TARGET-50-PAJNID** | 123bd4c3-6e36-4514-8d06-9f1f408cd1aa | 15751199-d931-5762-b206-b5d4c7043327 | NaN | NaN | NaN | NaN | NaN |
| **TARGET-50-PAJMWX** | 98d1ecc9-72b1-4dbb-83aa-aceac2621beb | ec8a85cd-2705-5c37-acdd-f79b722e7cca | NaN | NaN | NaN | NaN | NaN |
| **TARGET-50-PAJLMJ** | 1a6e21eb-01ca-5572-8260-178b28cd7428 | NaN | NaN | NaN | NaN | NaN | NaN |

As can be seen in the figure above, this dataframe maps each patient submitter ID to one or more aliquot IDs. Here the submitter ID is the index column. First, only submitter IDs belonging to TCGA are filtered out from the cnv_aliquotes dataframe and subsequently a submitter ID to aliquot ID mapper is created using these two dataframes. Finally, each column name in the CNV data (aliquot ID) is replaced by its corresponding submitter ID.

Next, two unnecessary columns (Gene ID, Cytoband) are removed. Additionally, the CNV data has a gene ID => submitter ID mapping of size (19729, 509) and undergoes a transpose to arrive at the correct configuration of submitter ID => gene ID mapping with a size of (509, 19729).

Next, a new column by the name of submitter_id is added to the table and made the index. The duplicate rows (same submitter ID) are removed while keeping the first occurrence.

Next, the value -1 is replaced with 2 through the dataframe as negative values are not allowed when pretraining. Finally, as stated in the paper, this CNV data undergoes further preprocessing by filtering only the top 2000 high-variance genes and subsequently performing a Chi-Squared analysis on the data. Chi-squared analysis was performed using k = {128, 256} and the best results were produced with k = 256.

| Number of features (k) | ROC-AUC Score |
|---|---|
| 128 | 0.829 ± 0.042 |
| 256 | 0.88 ± 0.018 |

## SM1.3 Clinical Data

### *1.3.1 Clinical Data Procurement*

The clinical data was procured by the R script provided in [5], using the TCGABioLinks R Package. First, the GDC portal was queried for all TCGA project IDs, using which clinical data for each of the 33 TCGA Projects were collected and merged into a single table. This was written onto a TSV file for preprocessing which is entailed in the section below.

### *SM1.3.2 Clinical Data Preprocessing*

The original dataset size of clinical data is (11315, 141). First, an information-based filter is applied to filter out only the features that are known to be informative. Next, an additional filter is applied to select only those features that have missing values below a certain threshold, bringing the dataset size to (11315, 14). Then the following set of sanity checks are performed in sequence –

- In all patients, race values if null are replaced by the corresponding ethnicity values. Next, patients that have race = ‘white’ and ethnicity = ‘hispanic or latino’ are replaced with race = ‘hispanic or latino’. Finally, the ethnicity column is removed. This is because we don’t require both race and ethnicity columns.
- Next, all patients that don’t have vital status recorded are filtered out.
- Next, patients that have both days_to_death and days_to_last_follow_up missing are removed as patients must have at least one of these recorded.
- Next, patients that are alive but are missing days_to_follow_up are removed. Similarly, patients that are dead but are missing days_to_death are also removed.

- Next, alive patients that have non-null values for days_to_death or dead patients that have non-null values for days_to_last_follow_up are both corrected as these are anomalous records.
- Next, patients that have days_to_last_follow_up as negative values are removed as well. Finally, only patients belonging to the TCGA-COAD project are preserved to arrive at a final dataset size of (458, 13).

Clinical data is made up of categorical variables and is hence encoded using a LabelEncoder. A dummy class called “NAN_VAL” is included to encode all the null values.

## SM2. Other Feature Reduction Techniques

As mentioned in the paper, a few other feature reduction and feature selection techniques were explored (with TabNet) on RNA-Seq data during the course of this study. However, none of them provided better results as compared to our three standard feature selection methods.

### SM2.1 Laplacian Score

Laplacian Score is a feature selection technique that provides a score to each feature that reflects its locality-preserving power. Laplacian Score was used as a feature reduction technique and selected a total of 74 features.

| Hyperparameter Name | Value |
|---|---|
| Metric | Euclidean |
| Neighbour_mode | knn |
| Weight_mode | heat_kernel |
| k | 5 |
| t | 1 |

See this notebook for the complete implementation. The results obtained were as follows –

| ROC-AUC | Standard Deviation |
|---|---|
| 0.711 | 0.02 |

## SM2.3 Autoencoders

Autoencoders were used as a feature reduction technique in this study. The encoding layer of an autoencoder was used to reduce the data into a more compact, representative dataset with the appropriate number of features. We reduced the dataset to 128 features as the output of the encoder layer. See this notebook for the complete implementation. The results obtained were as follows –

| ROC-AUC | Standard Deviation |
|---|---|
| 0.695 | 0.035 |

## SM2.4 Regularisation using Linear SVC

The concept of regularization is used here as a means to reduce overfitting and thereby select important features from a base estimator. The estimator used here is the linear support vector machine (Linear SVC). Linear SVC is an algorithm that attempts to find a hyperplane to maximize the distance between classified samples. The SelectFromModel which is a scikit-learn compatible API is a meta-transformer that is used for selecting important features(based on weights) from the base estimator, here Linear SVC. See this notebook for the complete implementation. The results are -

| ROC-AUC | Standard Deviation |
|---|---|
| 0.769 | 0.011 |

# SM3. Other Results

## SM3.1 Results with a t-test p-value of 0.001

| Model Name | Feature Selection | # of Genes | ROC AUC | Standard Deviation |
|---|---|---|---|---|
| Logistic Regression (l2 penalty) | Lasso | 80 | 0.721 | 0.059 |
| | PCA | 199 | 0.512 | 0.029 |
| | **T-Test** | **28** | **0.733** | **0.06** |
| Neural Network | Lasso | 80 | 0.528 | 0.091 |
| | PCA | 199 | 0.539 | 0.116 |
| | T-Test | 28 | 0.647 | 0.087 |
| EBMs | Lasso | 80 | 0.581 | 0.075 |
| | PCA | 199 | 0.534 | 0.06 |
| | T-Test | 28 | 0.699 | 0.083 |
| KNN | Lasso | 80 | 0.55 | 0.09 |
| | PCA | 199 | 0.553 | 0.089 |
| | T-Test | 28 | 0.572 | 0.073 |
| XGBoost | Lasso | 80 | 0.581 | 0.058 |
| | PCA | 199 | 0.579 | 0.055 |
| | **T-Test** | **28** | **0.727** | **0.043** |
| TabNet | Lasso | 80 | 0.729 | 0.031 |
| | PCA | 199 | 0.73 | 0.047 |
| | **T-Test** | **28** | **0.819** | **0.047** |
| **TabNet Multimodal** | **T-Test+Chi Squared** | **289 Features (28+256+5)** | **0.84** | **0.047** |

It can be observed that XGBoost performs better on a limited subset of features, while the best-performing model while using 230 features (Logistic Regression) ends up with a higher standard deviation.

## SM3.2 Results with different sizes of data

Further experiments involving different sizes of the training (labeled) dataset were tested to further understand the performance of the approach. The TabNet model was fine tuned to the task of vital status estimation with the percentages of the training data varying from 50% to 90%. The T-Test feature reduction technique was chosen for these experiments due to promising ROC-AUC scores obtained earlier in this study. The table below provides the average ROC-AUC score and standard deviation of the approach on cross validation:

| Percentage of Training data | ROC AUC Score | Standard deviation |
|---|---|---|
| 90% | 0.8863 | 0.04 |
| 80% | 0.8379 | 0.04 |
| 75% | 0.8248 | 0.03 |
| 50% | 0.77 | 0.01 |

## SM3.3 Multimodal Results with other models(apart from Tabnet):

The efficacy of self-supervised learning using multiple modalities of data were further experimented with the following set of models:

1. TabTransformer [6] notebook link
2. FT-transformer [7] notebook link
3. SAINT [8] notebook link

| Model | ROC AUC Score | Standard deviation |
|---|---|---|
| TabTransformer | 0.7201 | 0.088 |
| FT-Transformer | 0.5606 | 0.018 |
| SAINT | 0.5 | 0.0 |

# SM4. Feature Importances

## SM4.1 Logistic Regression Model

The table below lists the top 10% of the genes in descending order of feature importance scores according to the Logistic Regression model. For a complete, comprehensive list of all the genes, look here. SHAP was used for this.

| Gene | Importance | Cumulative Importance |
|---|---|---|
| DEFA6 | 0.03514869327 | 0.03514869327 |
| RBM3 | 0.03431965057 | 0.06946834383 |
| XBP1 | 0.02961725306 | 0.09908559689 |
| PRDX3 | 0.02804139097 | 0.1271269879 |
| S100P | 0.02786050019 | 0.1549874881 |
| LRRC59 | 0.02772631537 | 0.1827138034 |
| NOS2 | 0.02725918335 | 0.2099729868 |
| REG4 | 0.02507048489 | 0.2350434717 |
| GSR | 0.02364526878 | 0.2586887404 |
| TMED2 | 0.02339263615 | 0.2820813766 |
| DEFA5 | 0.02278406941 | 0.304865446 |
| DCAF7 | 0.02224128434 | 0.3271067303 |
| CCL25 | 0.02133595941 | 0.3484426897 |
| SARAF | 0.02088251315 | 0.3693252029 |
| CCDC47 | 0.01861536879 | 0.3879405717 |
| ARHGAP4 | 0.01822998547 | 0.4061705571 |
| CANX | 0.0181282279 | 0.424298785 |
| SEL1L3 | 0.0180933868 | 0.4423921718 |
| PROCR | 0.01806196863 | 0.4604541405 |
| GZMB | 0.01719370191 | 0.4776478424 |
| FDFT1 | 0.01717189031 | 0.4948197327 |
| TMEM98 | 0.01701233686 | 0.5118320696 |
| EMP2 | 0.01554617898 | 0.5273782485 |

## SM4.2 TabNet

The table below lists the top 10% of the genes in descending order of feature importance scores according to the TabNet model. For a complete, comprehensive list of all the genes, look here.

| Gene | Importance | Cumulative Importance |
|---|---|---|
| MAD2L1 | 0.1052165025 | 0.1052165025 |
| RBM3 | 0.1028368954 | 0.2080533979 |
| REG4 | 0.05043647845 | 0.2584898764 |
| CCNB1 | 0.04760210815 | 0.3060919845 |
| S100P | 0.04541071219 | 0.3515026967 |
| PGM2 | 0.04063063183 | 0.3921333285 |
| ASRGL1 | 0.03907442232 | 0.4312077508 |
| DEFA5 | 0.0327976978 | 0.4640054486 |
| ERI1 | 0.03118471 | 0.4951901586 |
| GSPT1 | 0.028256354 | 0.5234465126 |
| AKAP8L | 0.02791061087 | 0.5513571235 |
| ENO3 | 0.02666976851 | 0.578026892 |
| CCL25 | 0.02603089604 | 0.6040577881 |
| DEFA6 | 0.02595921227 | 0.6300170003 |
| LRIG3 | 0.02576401985 | 0.6557810202 |
| SURF6 | 0.02497691412 | 0.6807579343 |
| FLOT1 | 0.02197363913 | 0.7027315734 |
| ARHGAP4 | 0.02119532392 | 0.7239268973 |
| TTC21A | 0.0201685988 | 0.7440954961 |
| TAF1C | 0.01831368923 | 0.7624091854 |
| HDAC7 | 0.01763059014 | 0.7800397755 |
| PRR3 | 0.01708470858 | 0.7971244841 |
| INTS3 | 0.01637139096 | 0.8134958751 |

## SM5. Hyperparameter Tuning

Optuna is the main hyperparameter tuning framework used in this study. It relies on setting the workflow as an objective function and the evaluation metric as a parameter to optimize. It is known to perform efficient hyperparameter search, can run in parallel and has a model-agnostic API.

Optuna was employed to work on TabNet due to the variety of hyperparameters that can be tuned on the model, namely:

1. Learning Rate
2. Patience (number of waited epochs for early stopping)
3. Scheduler Patience (the number of waited epochs for the lr scheduler used)
4. Lambda_Sparse ( the importance of TabNet's internal sparsity loss)
5. Optimizer Parameters
    a. Weight Decay
    b. Momentum
    c. Gamma

For all the models used in this study, an Optuna study was created and run for 5 trials in total where the hyperparameters were varied to optimize the average cross-validation ROC-AUC score of the model considered.

The sections below, 2.1 and 2.2 entail the hyperparameters and the plots that were generated by Optuna in this study for the unimodal (T-Test feature reduction technique) and multimodal TabNet implementations.

## SM5.1 Hyperparameter tuning for TabNet - RNA-Seq

As explained in the introduction to this section, Optuna was run on a total of 5 trials, which yields the following hyperparameters:

| Hyperparameter Name | Value |
|---|---|
| Learning Rate | 0.3157 |
| Lambda_sparse | $4.882 \times 10^{-7}$ |
| Patience | 574 |
| Scheduler Patience | 690 |
| Weight Decay | 0.0001 |
| Momentum | 0.39 |
| Gamma | 2 |

The following parallel coordinate plot was obtained for the 5 trials that Optuna was used to tune the hyperparameters:

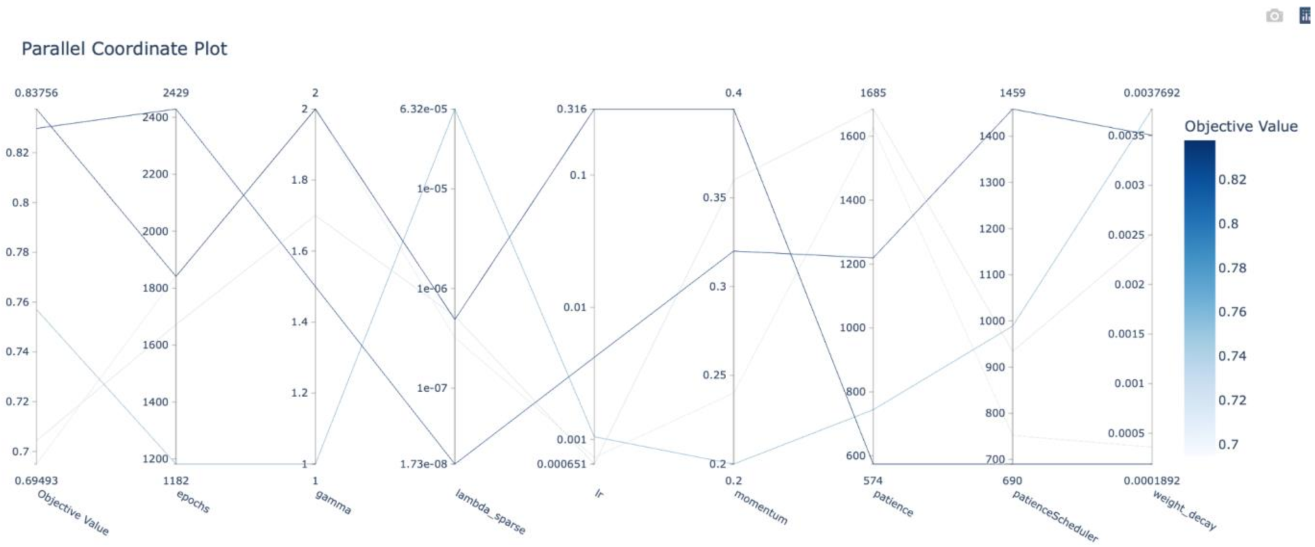

In the above parallel coordinate plot, each of the line segments represent the Optuna trials that were executed, with their corresponding value of the hyperparameter being the intercepted by the line segment in each of the hyperparameter axes.
The best trial (line segment with Objective Value of 0.83756) from the above plot has the hyperparameters that were finally accepted to infer.

A hyperparameter importance plot was also plotted to understand the importance of the hyperparameters tuned with Optuna.

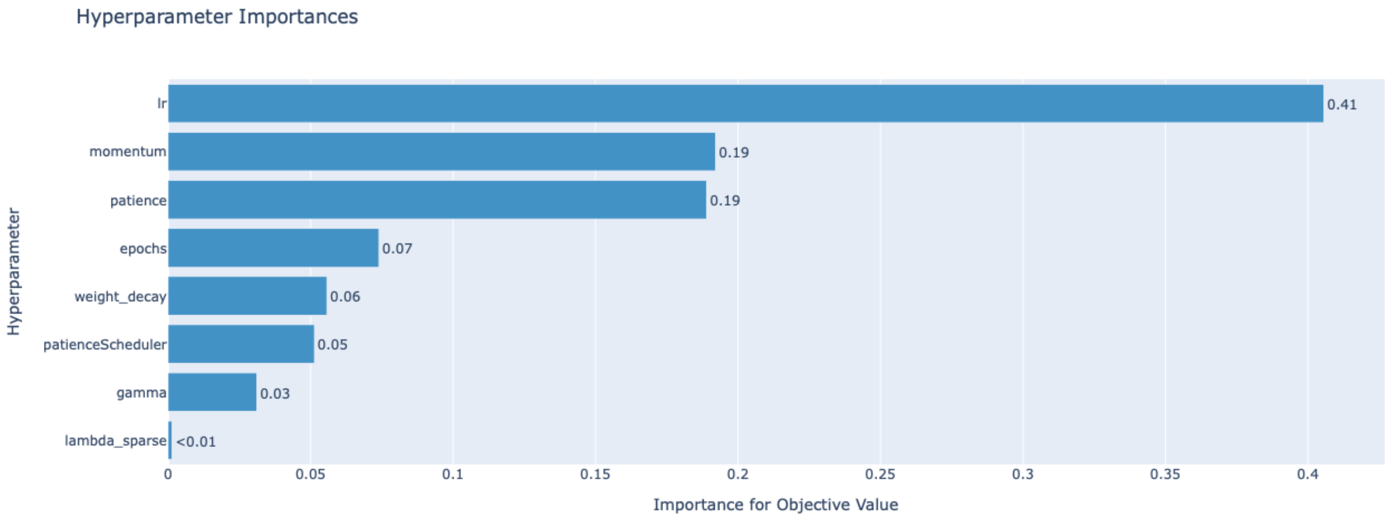


The above importance plot indicates the relative importance of the hyperparameters varied by Optuna. It can be inferred that the learning rate has the highest influence on the objective value (model’s average ROC-AUC Score). It can also be inferred that lambda_sparse, the parameter that corresponds to the sparsity-inducing regularization of the TabNet model, has the least impact on the objective value.

## SM5.2 Hyperparameter tuning for TabNet - Multimodal

The following hyperparameters were obtained after Optuna completed 5 trials with the TabNet model on the multimodal data.

| Hyperparameter Name | Value |
|---|---|
| Learning Rate | 0.057 |
| Lambda_sparse | $3.909 \times 10^{-6}$ |
| Patience | 1080 |
| Scheduler Patience | 1332 |
| Weight Decay | 0.00355 |
| Momentum | 0.04 |
| Gamma | 1.4 |
| epochs | 1884 |

The following parallel coordinate plot was obtained post hyperparameter tuning.

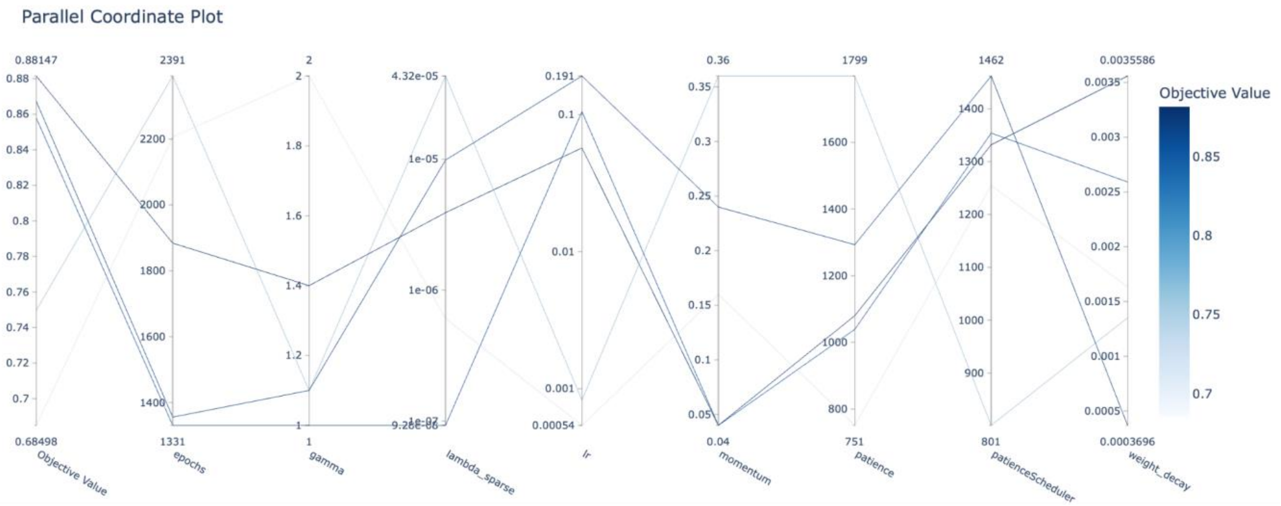

As explained earlier the parallel plot gives us a view of the hyperparameters used for each trial based on their objective value. It can be observed here that low values of momentum were seemingly preferred.

The corresponding hyperparameter importance plot was also obtained:

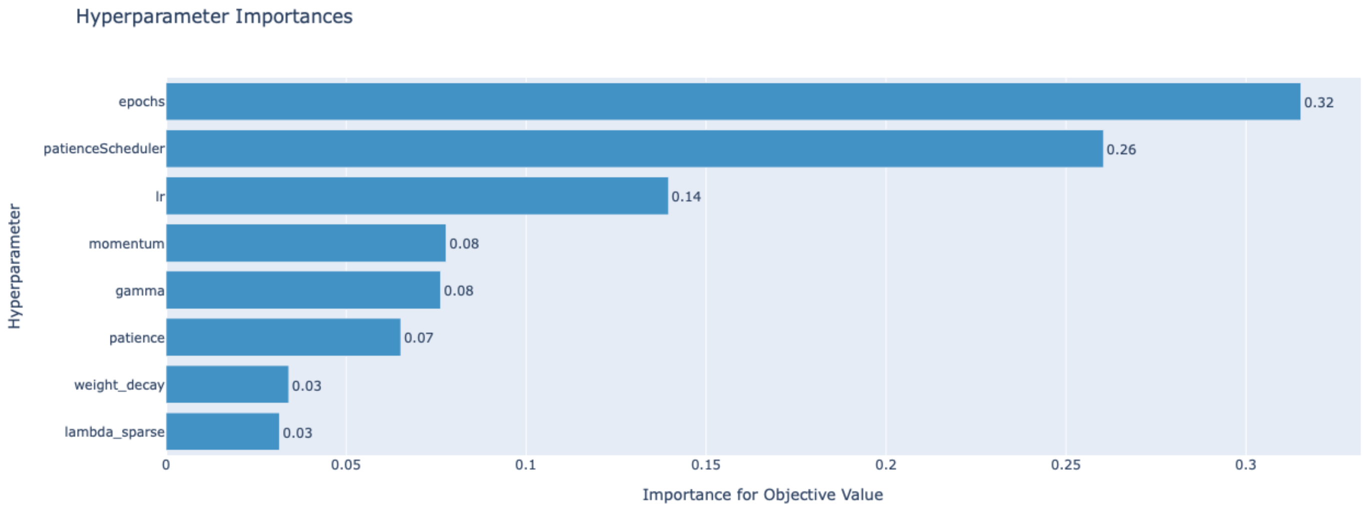


It can be inferred that the number of epochs influences the objective value greatly. The patienceScheduler and lr have the next highest importance for the objective value. It is also observed that similar to unimodal TabNet, the sparsity-inducing regularization has the least impact on the objective value.

## References

[1] Grossman, Robert L., Heath, Allison P., Ferretti, Vincent, Varmus, Harold E., Lowy, Douglas R., Kibbe, Warren A., Staudt, Louis M., "Toward a Shared Vision for Cancer Genomic Data", *New England Journal of Medicine*, 2016, vol. 375:12, pp. 1109-1112.

[2] COAD Alive, Data Repository, https://drive.google.com/file/d/1kUhoattd0Tllsj9hBvYwJ_v2OrlMPOwL/view?usp=share_link, last accessed: 13 January 2023.

[3] COAD Dead, Data Repository, https://drive.google.com/file/d/11nwIUomPPCcnFJchLYfEtXCC5EUJBJpc/view?usp=share_link, last accessed: 13 January 2023.

[4] Adenomas and Adenocarcinomas, Data Repository, https://drive.google.com/file/d/1HXsu6kE5Pc6o124Bb3d9yX2398Rg1vE-/view?usp=share_link, last accessed: 13 January 2023.

[5] Vale-Silva, Luís A., and Karl Rohr. "Long-term cancer survival prediction using multimodal deep learning." *Scientific Reports*, 2021, vol. 11.1, pp. 1-12.
[6] Huang X, Khetan A, Cvitkovic M, Karnin Z. Tabtransformer, “Tabular data modeling using contextual embeddings”, *arXiv preprint*, Dec 2020, arXiv:2012.06678.
[7] Gorishniy Y, Rubachev I, Khrulkov V, Babenko A., “Revisiting deep learning models for tabular data”, *Advances in Neural Information Processing Systems,* Dec 2021, vol. 34 pp. 18932-43.
[8] Somepalli G, Goldblum M, Schwarzschild A, Bruss CB, Goldstein T., “Saint: Improved neural networks for tabular data via row attention and contrastive pre-training”, *arXiv preprint* Jun 2021. arXiv:2106.01342.
